# A novel computational approach to pain perception modelling within a Bayesian framework using quantitative sensory testing

**DOI:** 10.1038/s41598-023-29758-8

**Published:** 2023-02-23

**Authors:** Armin Drusko, David Baumeister, Megan McPhee Christensen, Sebastian Kold, Victoria Lynn Fisher, Rolf-Detlef Treede, Albert Powers, Thomas Graven-Nielsen, Jonas Tesarz

**Affiliations:** 1 Department of General Internal Medicine and Psychosomatics, University Hospital Heidelberg, Im Neuenheimer Feld 410, 69120 Heidelberg, Germany; 2 Center for Neuroplasticity and Pain (CNAP), Department of Health Science and Technology, Aalborg University, Aalborg, Denmark; 3 Mannheim Center for Translational Neuroscience (MCTN), Heidelberg University, Heidelberg, Germany; 4 Department of Psychiatry, Yale University School of Medicine, New Haven, CT USA

**Keywords:** Neuroscience, Cognitive neuroscience, Computational neuroscience, Sensory processing, Rheumatology, Fibromyalgia

## Abstract

Pain perception can be studied as an inferential process in which prior information influences the perception of nociceptive input. To date, there are no suitable psychophysical paradigms to measure this at an individual level. We developed a quantitative sensory testing paradigm allowing for quantification of the influence of prior expectations versus current nociceptive input during perception. Using a Pavlovian-learning task, we investigated the influence of prior expectations on the belief about the varying strength of association between a painful electrical cutaneous stimulus and a visual cue in healthy subjects (N = 70). The belief in cue-pain associations was examined with computational modelling using a Hierarchical Gaussian Filter (HGF). Prior weighting estimates in the HGF model were compared with the established measures of conditioned pain modulation (CPM) and temporal summation of pain (TSP) assessed by cuff algometry. Subsequent HGF-modelling and estimation of the influence of prior beliefs on perception showed that 70% of subjects had a higher reliance on nociceptive input during perception of acute pain stimuli, whereas 30% showed a stronger weighting of prior expectations over sensory evidence. There was no association between prior weighting estimates and CPM or TSP. The data demonstrates relevant individual differences in prior weighting and suggests an importance of top-down cognitive processes on pain perception. Our new psychophysical testing paradigm provides a method to identify individuals with traits suggesting greater reliance on prior expectations in pain perception, which may be a risk factor for developing chronic pain and may be differentially responsive to learning-based interventions.

## Introduction

Modern approaches conceptualize perception as statistical inference of unknown environmental states comprising sensory information and internal prior expectations^1–3^. This can be formalized within a Bayesian framework, where prior and sensory information are integrated yielding combined Gaussian probability distributions that represent expectations of states (mean) and their uncertainty (precision)^2–4^. This Bayesian understanding has been already validated across audition, vision and somatization^5–13^.

In alignment with this, pain perception can also be conceptualized as Bayesian inference, where prior information and sensory input are integrated to infer the noxious properties of a stimulus^3,14–17^. Prior information on pain can be acquired in a variety of ways—i.e. conditioned learning, observation or neurocognitive processes^17,18^. Its influence on processing of sensory input suggests a hierarchical system with top-down predictions and their uncertainties integrated and updated by ascending nociceptive information and precision-weighted prediction errors^19^. For example, the prospect of more severe pain leads to increased pain perception, whereas the expectation of less pain can lead to pain relief, as placebo and nocebo research demonstrated^20–24^. In this way, a number of endogenous control phenomena can be explained that cannot be captured by conventional quantitative sensory measures of pronociceptive and antinociceptive mechanisms, as quantified by temporal summation of pain (TSP) or conditioned pain modulation (CPM)^25^.

This is of clinical relevance: pain represents an inference about the state of a body defect. In some cases, it becomes pathologically persistent in the form of chronic pain. This state likely results from an interaction between peripheral nociceptive inputs and aberrant central processes that amplify and maintain the pain^19^. An excessive and irrefutable belief that the defect persists could further bias perception during chronification (a “self-fulfilling prophecy”)^26^. Such subconsciously self-reinforcing effects may facilitate the transition from acute to chronic pain. Disentangling the weights attended to prior and sensory information during pain perception could open promising possibilities to more thoroughly understand the complexity of pain on an individual basis^27–29^. Bayesian learning models, coupled with an experimental learning paradigm for the introduction and testing of prior influences, have the ability to estimate the influence of prior and sensory information during perception^17,25^. One implementation of such model is the Hierarchical Gaussian Filter (HGF)^30^. The HGF is a recent derivation of one-step update equations from Bayesian principles based on a hierarchical generative model of the environment and its (in)stability^30,31^. Although, such computational methods exist, no suitable psychophysical paradigms have been developed for the quantification of prior and sensory information for the perception of nociceptive stimuli.

The purpose of this study was (1) to develop a nociceptive predictive processing (NPP)-task and use computational learning models to quantify the weighting of prior beliefs over observed sensory input during pain perception at the individual level, and (2) to investigate whether the weighting of prior beliefs versus sensory evidence, and common central pain sensitization processes (TSP and CPM) at spinal and supraspinal levels, have a common neurobehavioral basis or whether the weighting is an independent neurophysiological phenomenon worth studying separately. It is hypothesized that HGF can be used to describe the integration of sensory evidence with prior beliefs and quantify their influences during the perception of pain.

## Methods

### Sample recruitment and characterization

Seventy healthy participants, with a mean age of 24.0 ± 4.7 and a gender proportion of 46% women, were recruited through notices at Aalborg University, University College Nordjylland, on public noticeboards in Northern Jutland and on social media. Participants were included if they were healthy, aged 18–60 years and could speak, read, and understand English or Danish. Individuals were excluded in the case of pregnancy, current substance, opioids, antipsychotics, and benzodiazepines misuse and previous or current neurological, musculoskeletal, rheumatic, malignant, inflammatory, or mental illnesses. Also, volunteers with current or prior chronic pain conditions were excluded. The recruitment and conduction of this study was designed to comply with the Declaration of Helsinki and the protocol was approved by the North Denmark Region Committee on Health Research Ethics (VN-20180078).

### Experimental procedure

Each participant was informed about the study in a meeting prior to the experimental session. The session began with an introduction by the investigator and obtaining informed consent. Firstly, the demographic questionnaire was completed. Then, the mechanical pain detection protocol (pin-prick) was performed on both arms over the testing site for the NPP-task (volar forearm). Afterwards, the NPP-task protocol was conducted on the non-dominant arm. The NPP-task was described briefly by the investigator. All other instructions were presented on the monitor during the protocol. The investigator was present in the room but did not interact with the participant unless necessary. After the NPP-task, the mechanical pain detection procedure was performed a second time to investigate whether the NPP-task induced any sensitization of the test area. Then, the thresholds for perceived pain detection and pain tolerance (PDT and PTT), as well as temporal summation (TSP) and conditioned pain modulation (CPM), were assessed on the legs by cuff algometry. The participants filled out the Dissociative Experience Scale (DES) and the Cardiff Anomalous Perceptions Scale (CAPS) to assess whether the recruited sample of healthy subjects shows any signs of abnormalities in their perceptual behavior and whether a variability in weighting of prior versus sensory evidence is associated with dissociative experiences or anomalous perceptions in general (see supplementary material for more details)^32,33^.

### Mechanical pain detection protocol

To investigate potential changes in sensation at the test area, the mechanical detection threshold was measured using seven weighted pinprick stimulators (8–512 mN). The threshold was determined for the test and control arm (volar forearm) both before and after the NPP-task. The intensity (represented by the exerted force of the individual pinpricks in mN), was applied in an alternating ascending and descending manner until three “Yes” (a pricking sensation was felt) and two “No” (no pricking sensation was felt) responses were obtained from the participant^34^.

### Nociceptive predictive processing task

Based on a computational modelling approach of learning under uncertainty^30,31^, and inspired by the Conditioned Hallucination task from Powers et al.^35^, we developed a NPP-task assessing the relative weight of the prior versus the sensory input during perception of nociceptive stimuli. All nociceptive stimuli during the NPP-task were induced by bipolar cutaneous electrical stimulation to the forearm causing an intensity-dependent sensation from a non-painful to a painful pricking sensation. The NPP-task essentially comprised three steps: In the first step, maximum likelihood threshold estimation procedures (QUEST)^36^ were used to determine the individual perceptual threshold for nociceptive stimuli. In the second step, aberrant pain perceptions were elicited using a probabilistic Pavlovian conditioning paradigm (see below). For this purpose, all participants were asked to report whether they felt an electrical stimulus as a pricking sensation during the stages of the paradigm. The conditioning stage comprised of a high percentage of trials with intensities slightly above the perceptual threshold along a visual conditioning cue. Subsequently, the testing stage was comprised of a gradual decrease in association strength presenting sub-threshold intensities along with a progressive increase in the rate of zero-intensity trials. In the third step, the perceptual learning and unlearning of this association were modelled using a Hierarchical Gaussian Filter (HGF) model and the relative weight ($\upnu$) of the prior versus the sensory input during perception was estimated.

**Figure 1:**
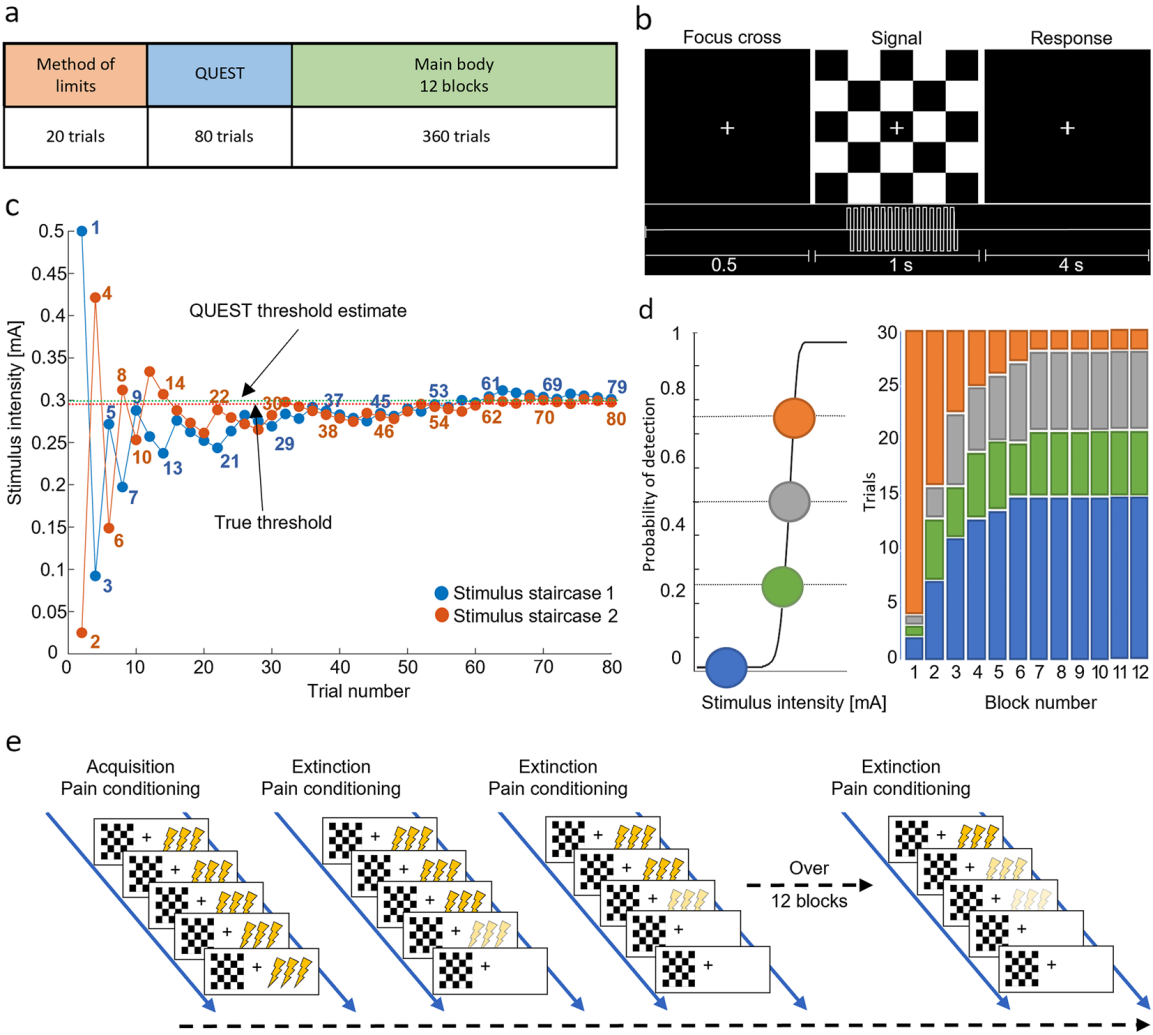
Nociceptive predictive processing task (NPP-task): The NPP-task was developed for the measurement of participant’s behavior during the administration of nociceptive stimuli via electrical stimulation with varying strengths in association between the nociceptive stimuli and a visual cue. (**a**) NPP-task structure: The task includes sensory threshold detection (methods of limits and QUEST) and the Pavlovian conditioning and testing (main body). (**b**) Trial structure: A single trial is comprised of an initial presentation of a focus cross, a subsequent checkerboard presentation with electrical stimulation (1 s duration) and a response time window (4 s) for the participant to indicate whether the stimulation was felt or not. (**c**) QUEST threshold estimation: Example of a QUEST run with two stimulus staircases (blue and orange lines) and associated stimulus intensities (blue and orange dots). The stimuli from both staircases are administered interchangeably with numbers (in the matching color of the staircases) indicating the order of presentation (some numbers are omitted for the sake of readability). The final estimated threshold after the QUEST run (green dotted line) and the hypothetical true threshold (red dotted line) are shown. (d) Psychometric curve and trial types: Example graph of a psychometric curve based on QUEST threshold estimates with derived trial types for the subsequent Pavlovian conditioning and testing. Each trial type is supposed to be detected with an expected rate (75%, 50%, 25% and 0%, presented in orange, grey, green and blue color, respectively). The distribution of different trial types administered during the task is shown in the scheme to the right. A set of 30 trials is organized in one block. Each block has a characteristic proportion of trial types. (**e**) Probabilistic Pavlovian conditioning: The conditioning paradigm ensures an introduction of a prior—visual cue conditioned to a nociceptive electrical stimulus—during an “Acquisition” phase, and the subsequent testing of participants perception with decreasing probabilities of high intensity trials during an “Extinction” phase; NPP-task—nociceptive predictive processing task; mA—milliamperes; s—second.

The experimental paradigm was comprised of the determination of the nociceptive threshold (method of limits and QUEST) and probabilistic Pavlovian conditioning with a visual cue (main body of the NPP-task; Fig. 1a) as inspired and utilized in the works of Powers et al.^35,37,38^.

All stimuli were presented in trials and participants’ responses were recorded with MATLAB Psychtoolbox 3.0. A visual fixation cross was present throughout each trial, white on a black background and the visual conditioning stimulus consisted of a checkerboard image (Fig. 1b). Visual stimuli were presented concurrently with target electrical stimuli, if present, for one second.

#### Determination of the nociceptive threshold

Stimulus intensities were determined using the QUEST maximum-likelihood procedure for threshold determination (Psychtoolbox 3.0, available at: http://psychtoolbox.org/). The QUEST is a Bayesian adaptive psychometric method used to determine individual stimulus intensities eliciting sensory perceptions with a sufficient degree of perceptual uncertainty in the individual^36^. First, the QUEST assesses the threshold for electrical stimuli for which the participants are 75% likely to report detection. This is accomplished via two independent, 40-trial-long ascending and descending series of stimulus intensities (stimulus staircases) determined by the QUEST program based upon participant responses (Fig. 1c) with a subsequent derivation of the mean threshold value from the posterior distribution of threshold intensities^35^. Prior to the QUEST, a coarse threshold paradigm is conducted using a method of limits approach with five detections to determine a starting value for the QUEST. After the QUEST paradigm, individual psychometric curves were fitted to participant responses using a log-Weibull curve from which stimulus intensities, at which participants are 75%, 50% and 25% likely to detect stimulus presentations, were derived. The beta, gamma and delta parameters of the log-Weibull model for the psychometric curve were set to values of 3.5, 0.01 and 0.01, respectively^35^.

#### Probabilistic Pavlovian conditioning

During the main body of the NPP-task (Fig. 1a), participants were implicitly taught the association between the target electrical stimulus and a concurrently presented visual conditioning cue stimulus (checkerboard image; Fig. 1b). Subsequently, they were tested on the strength of this association over the course of 12 blocks of 30 trials each (Fig. 1d). Following each trial, participants were asked to indicate whether the target electrical stimulus was present (in form of a pricking sensation) using a button press. The likelihood of threshold-level electrical stimulus presentations decreased non-linearly over the course of the 12 blocks with concomitant overrepresentation of subthreshold and zero-intensity trials (Fig. 1e). Trial-type relative likelihoods were determined by block number, and trial type presentation was pseudorandomized within blocks. To avoid habituation or sensitization to the electrical stimuli, the presentations were separated by a fixed time of 4 s, in which the participants were allowed to give a response. Participants were prompted to take a short break of up to 2 min after every three blocks.

#### Computational modelling using hierarchical Gaussian filters

A Bayesian framework with a Hierarchical Gaussian Filter (HGF) was used to model the integration of sensory input with prior beliefs during perception to yield in a posterior belief about the states causing the sensory inputs^30,31^. This modelling approach is based on the theory of agents that learn about the world by perceiving sensory inputs, constantly updating their internal model of hidden states that caused these inputs, and taking actions based on their new beliefs^2,4,39^. As inspired by Behrens et al. 2007, hidden states of the world are represented by a generative model of hierarchically coupled Gaussian random walks that evolve in time^40^. The HGF approach allows for the utilization of a dynamic and adaptive learning rate, which tackles the process of learning under uncertainty in a changing environment. Each higher level in the hierarchy represents the dynamic structure of the world and each step size of the random walk is influenced by the next-higher state in the hierarchy. By inverting this generative model with variational approximation, we can derive update rules for the expectation of hidden states that the agent has at any given time. Furthermore, the introduction of prior parameters that govern the coupling between levels in the hierarchy, allows for the extraction of learning characteristics that vary between individuals and allows for learning that is subjectively optimal, but objectively erroneous—as is expected with strong priors. By fitting the HGF to our behavioral data from the NPP-task, a weighting parameter ($\upnu$) between prior expectations and sensory evidence could be extracted. More information about the HGF implementation is given below.

### Electrical stimulation

Due to ethical reasons, an intraepidermal electrode for the stimulation of A-δ fibers, as recommended in the work of Inui et al., could not be implemented in this study^41^. As a workaround, a bipolar electrode with two steel balls for skin contact (7 mm stimulation points at 28 mm distance) was secured 5 cm distal to the elbow on the volar forearm. The total signal duration was set to one second with a pulse train of 15 bipolar waves, with a frequency of 50 Hz, starting at 200 ms. There was no intensity (0 milliamperes; mA) before and after the pulse train. The pulse waves were rectangular as suggested by Inui et al., whereas the remaining parameters had to be adjusted (polarity, frequency and duration), to achieve a pricking like sensation upon stimulation^41^. For stimulus presentation, this signal was sent to the data acquisition board (National Instruments; Model: USB-6341) and further to a DS-5 (Digitimer, United Kingdom) electrical stimulation device with the attached electrode. The electrical signal was defined in MATLAB.

### Cuff algometry

A computerized pressure cuff system (Nocitech, Denmark) was used to investigate the perceived pain detection (PDT) and pain tolerance thresholds (PTT) as well as temporal summation of pain (TSP) and conditioned pain modulation (CPM)^42^. The protocol included a ramp to assess PDT and PTT on the dominant leg, a TSP paradigm on the dominant leg, a ramp to assess PDT and PTT on the non-dominant leg, and a CPM paradigm with the test stimulus on the dominant and conditioning stimulus on the non-dominant leg. The cuffs were placed over the widest portion of the gastrocnemius muscle on each lower leg with participants in a reclined long sitting position with a pillow under the knees. Participants were asked to rate the intensity of pain felt during the pressure application using an electronic visual analogue scale (VAS). The VAS score for pain ranged from 0 (no perceived pain) to 10 cm (maximum tolerable pain). The maximum cuff pressure was limited to 100 kPa and participants were able to immediately deflate the cuffs by pushing a button. Each subject was familiarized with the cuff algometry device during a training phase in the start of the session, with the subject seated and the cuff placed around the upper arm. Subjects were trained on how to use the electronic VAS and how to stop cuff inflation.

Ramps conducted to assess PDT and PTT used steadily increasing pressure (1 kPa/s) to a maximum of 100 kPa. The VAS was used by the subjects to provide continuous VAS ratings of their pain intensity during cuff inflation. When the VAS reached 1 cm, the corresponding cuff pressure was taken as the subject's PDT^42^. Once the pressure reached the subject's tolerance limit, they pushed the stop button on the VAS device to deflate the cuffs and this was taken as their PTT. For subjects who reached the maximum pressure of 100 kPa, this was taken as a conservative estimate of their PTT^43^.

TSP was assessed by ten cuff stimuli at PTT intensity with 1 s duration and 1 s interstimulus interval^42,44^. The participants were instructed to rate their pain from the first stimulus immediately on the VAS, then to adjust their rating up as necessary on subsequent stimuli without returning the dial to zero. The rating for the first stimulus was used as a normalization factor (subtracted from the VAS scores related to the 2nd to the 10th stimuli). The TSP-effect ratio was calculated as the average of the pain ratings of the last three pulses, divided by the average of the pain ratings during the first three pulses.

The CPM protocol included rapidly increasing pressure on the nondominant leg to 70% of PTT (conditioning stimulus), which then remained constant throughout. A steadily increasing pressure (as per initial PTT determination) was then applied to the dominant leg to capture the conditioned PTT (test stimulus). The CPM effect was calculated as the difference between the PTT of the dominant leg during conditioning and the PTT measured at baseline.

### Computational modelling

HGF modelling was used to quantify the influence of prior beliefs about sensory inputs on perception at the individual level. The HGF is a general Bayesian hierarchical model of learning that describes the participant’s perception or learning about environmental states under uncertainty and changing conditions^31^. The modelling framework is comprised of a perceptual model that maps the sensory input to hidden environmental states causing these inputs, and an observational model with decision noise that describes the participant’s responses based on the beliefs about the hidden states. The perceptual model describes the evolution of beliefs about the changing environmental states and their uncertainty. In our implementation, these states are represented with three coupled levels (*x*_1−3_) that evolve as Gaussian random walks. Each higher level is determining the uncertainty (variance) of the level directly underneath. The first level (*x*_1_) represents the belief about the presence of the sensory input at trial *k*, the second level (*x*_2_) is the belief about the cue-stimulus contingency and the third level (*x*_3_) represents the inferred volatility about the contingency of level 2. The evolution of the states over time can be represented with the posterior distributions:

1 $$p(x_{1} | x_{2} ) = s(x_{2} )^{{x_{1} }} (1 - s\left( {x_{2} } \right))^{{1 - x_{1} }}$$

2 $$p\left( {x_{2}^{\left( k \right)} |x_{2}^{{\left( {k - 1} \right)}} , x_{3}^{\left( k \right)} } \right) = {\mathcal{N}}\left( {x_{2}^{\left( k \right)} ; x_{2}^{{\left( {k - 1} \right)}} , \exp \left( {\kappa x_{3}^{\left( k \right)} + \omega } \right)} \right)$$

3 $$p\left( {x_{3}^{\left( k \right)} | x_{3}^{{\left( {k - 1} \right)}} , \vartheta } \right) = {\mathcal{N}}\left( {x_{3}^{\left( k \right)} ; x_{3}^{{\left( {k - 1} \right)}} , \vartheta } \right)$$

where ${x}_{i}^{(k)}$ is the environmental state at level *I* and trial *k*, $\omega$ represents a scaling factor that is independent of the third level, $\kappa$ determines the strength with which the estimated volatility affects the learning about the second level and $\vartheta$ represents a constant volatility of the third level. $s\left(x\right)$ is a sigmoid function:

4 $$s\left( x \right) = \frac{1}{{1 + {\text{exp}}\left( { - x} \right)}}$$

Given the equations above, variational approximation to ideal Bayesian learning yields in analytical equations that allow the estimation of the updates of posterior beliefs about state *x* at level *i* and trail *k* based on precision-weighted prediction errors from the level below (*i* *−* *1*).

**Figure 2:**
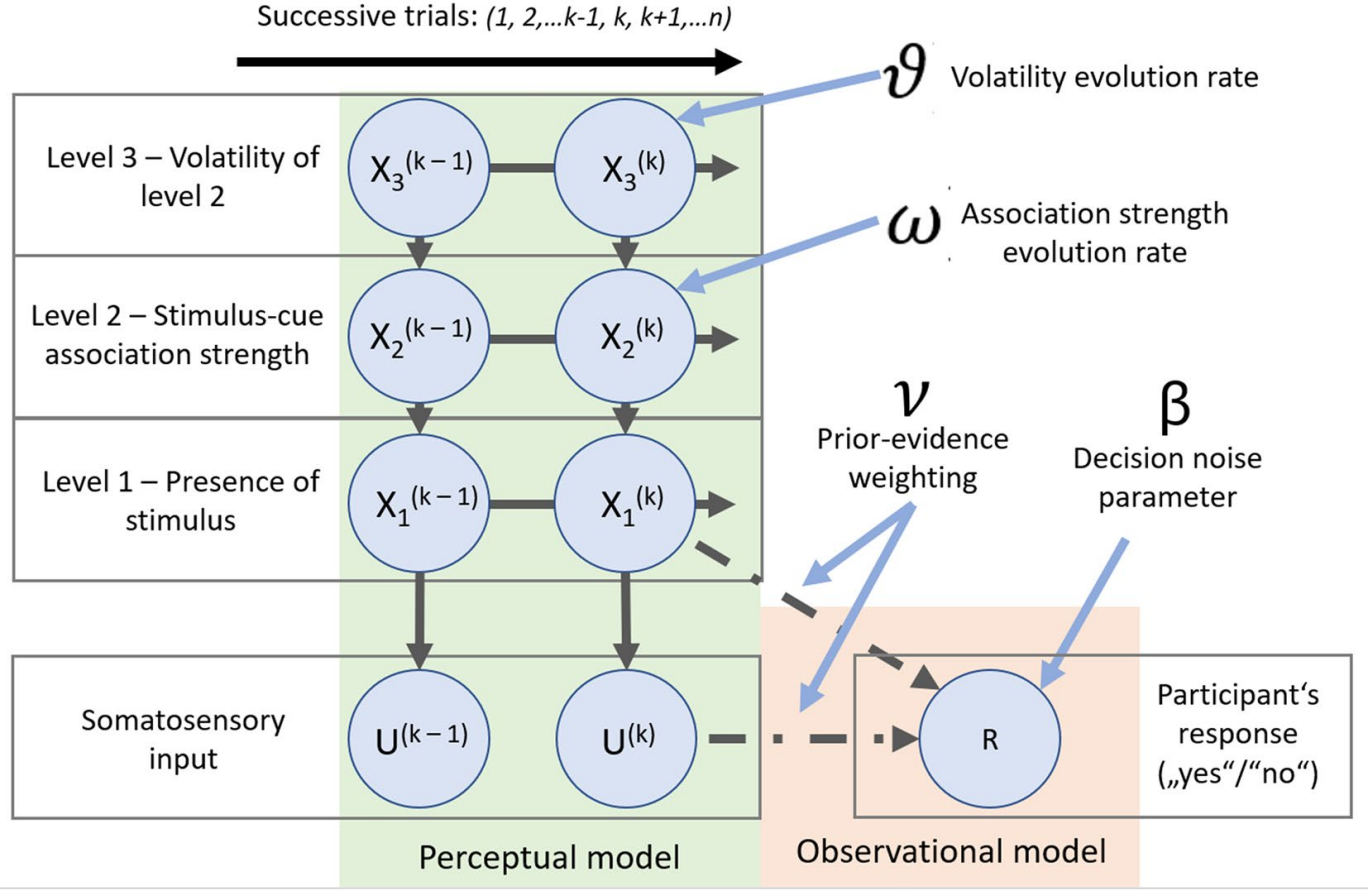
Modelling framework. The HGF is a Bayesian hierarchical model that describes the learning about a continuous uncertain quantity that changes over time through sequential input. The modelling framework consists of a perceptual and an observational model. The perceptual model maps the sensory inputs (U) to inferred hidden states (x_1−3_) of the world that are causing these inputs. In a three-level HGF implementation with behavioral data from the NPP-task, with conditioning between electrical stimulus and a visual cue, the levels describe the: (**1**) inferred belief about the presence of a stimulus at trial k, (**2**) belief about the association strength between electrical stimuli and the visual cue, (**3**) belief about the volatility of the association from level 2. The state of x at level i and trial k is influenced by its previous state k-1. Additionally, the levels are hierarchically coupled so that values of higher-level states influence the dynamics of the levels directly beneath. Furthermore, the scaling factors $\omega$ and $\vartheta$ affect the evolution of the progression of levels 2 and 3, respectively. The observational model incorporates information on the prior probability of an electrical stimulus and the observed signal intensity to yield a posterior probability of a stimulation. Introducing decision noise, represents by β, the probability of a “yes” response (R) (i.e., the stimulus was felt), can be described. The observational model allows for an individual variability in the weighting of prior beliefs and sensory evidence during belief update (parameter $\nu$). HGF—Hierarchical Gaussian Filter; NPP-task—nociceptive predictive processing task.

We modelled the learning about the association between a visual and nociceptive stimulus and aimed to extract the weighting between prior beliefs about incoming stimuli and actual sensory evidence during perception. For this, we implemented an observational model, in line with the work of Powers et al. using a very similar procedure in audition, where decision noise was applied to the probability of a detection^35^. The “belief” of the participant, i.e., the posterior probability of a stimulation, is given by the participant’s prior and the strength of the observed stimulation. This was formalized as the Bayesian posterior of a beta distribution:

5 $$belief = prior + \frac{1}{1 + v}\left( {observation - prior} \right)$$

where *observation* is the stimulus intensity of the trial, *prior* is the learning prior from the HGF’s level 1, and *ν* is a parameter indicating the relative weight of the prior vs the sensory input so that for values greater than 1 the prior has more weight than the observation and values lower than 1 indicate greater weighting of the observation. The logistic sigmoid is:

6 $$f\left( x \right) = \frac{1}{{e^{{\left( { - \beta *\left( {V_{1} - V_{0} } \right)} \right)}} }}$$

where β is a positive parameter that determines the slope, and *V*_*1*_ and *V*_*0*_ are the values of detection vs non-detection, respectively. In this study, there will be focus on the *ν*-parameter, given the likely importance of prior weighting in the perceptual computation of pain in chronic pain conditions. Here, $\upnu$ < 1 indicates a stronger weighting of prior expectations over sensory information, whereas $\upnu$>1 indicates a stronger weighting of sensory information over prior expectations. Figure 2 shows a schematic representation of the modelling framework implemented in this study.

In this study, we used the HGF modelling implementation from the TAPAS toolbox (Version 6.0.1, available at: http://www.translationalneuromodelling.org/tapas/) in the MATLAB language (MATLAB and Statistics Toolbox Release Version 5.0.0, The MathWorks, Inc., Natick, Massachusetts, United States)^45^. The initial parameter states of the HGF were set to default as defined in the HGF toolbox (v7.1).

During the fitting of the HGF, we observed issues achieving a fit for several participants, which was corrected by altering the initial state for the Ω-parameter, which forms part of the linking function between the second and third level of the HGF. A systematic search of parameter values from the initial prior of − 3 up to − 10 revealed optimal fit at an initial value of - 4.2, in that the model was able to converge on a fit for all participants but without inducing outliers in the *ν-*parameter or fit indices (LME/AIC). To prevent undue influence of outliers in the *ν*-parameter fit, a robust regression approach was undertaken (elaborated further below).

We compared the ability of the HGF implementation with the $\upnu$-parameter to fit and simulate the behavioral data with non-Bayesian models and without $\upnu$. For this, a simulation analysis and Bayesian model comparison was conducted. Please see the supplementary material for more details on the utilized methods and results.

### Statistical analysis

#### Inferential models

All statistical analyses were carried out in R (version 4.1.1), using Bayesian estimation in *brms* which acts as a high-level interface to *Stan* to implement a no-U-turn sampler variant of Hamiltonian Monte Carlo (HMC)^46–48^. For all models fitted, four chains were run using 5000 iterations at a warm-up of 1000. The models were specified with mildly informative priors to achieve regularization. Outcome parameters were estimated using Student’s t-distributions rather than Gaussian normal distributions for all models, which are more robust to outliers^49^. All predictors were scaled and centered. Two main models were fitted, with the specifications:

7 $$\gamma_{i } \sim {\text{t}}\left( {\mu_{i} , \sigma , {\upnu }} \right)$$

8 $$\mu_{i} = \alpha_{i} + { }\beta_{j} X_{j}$$

9 $$\alpha_{i} \sim {\text{Normal}}\left( {0,2.5} \right)$$

10 $$\beta_{j} \sim {\text{Normal}}\left( {0,2.5} \right)$$

11 $$\sigma \sim {\text{HalfCauchy}}\left( {0,1} \right)$$

12 $${\upnu } \sim {\text{Exponential}}\left( 1 \right)$$

where ${\gamma }_{i}$ is the outcome,${\alpha }_{i}$ the intercepts of pre- and post-task thresholds, ${\beta }_{j}$ a vector of coefficients for the vector of covariates ${X}_{j}$ (gender and QUEST threshold), $\sigma$ is the initial value of the scaling parameter, and $\upnu$ the degrees of freedom of the t (not to be confused with the $\upnu$-parameter from the HGF).

First, a robust regression model was fitted to assess whether the task induced systematic sensitization/habituation in the test area, with mechanical pain detection threshold assessed via pinprick as the outcome and pre- and post-task timepoint as the primary predictor. Additionally, gender and QUEST threshold were entered as covariates. The model was set up to estimate individual intercepts for pre- and post-task, with the assumption that an effect of arm by time indicates a systematic change in the test arm across the two measurement timepoints. The outcome of this regression model was not the perception of each stimulus during the task, but the response to mechanical stimulation before and after the task to ensure that task performance itself is not potentially confounded by systematic sensitization/habituation. So, we assessed MPT before and after the task itself, and stipulated that an effect of arm by time (one arm changes over time but the other does not), indicates potential sensitization/habituation across the sample.

Second, a robust regression model was fitted to assess whether the weighting of prior to likelihood in the NPP-task, instantiated in the *ν*-parameter, was linearly associated with TSP-effect, CPM-effect, PDT, PTT and QUEST threshold. The three parameters showed no evidence of multicollinearity and were therefore entered into the same model. Again, gender was entered as a covariate to control for its potential influence on outcome and predictors.

HMC chain convergence was assessed using the Rhat value, reliability of posterior quantiles was evaluated in the bulk- and tail-Effective Sample Size (ESS), and predictive accuracy was evaluated using the leave-one-out cross-validation information criterion (LOO-IC) to estimate the expected log predictive density^50,51^. Further, Bayesian R^2^ estimates (which define R^2^ based on the variance of estimated prediction errors) are provided^52^. To provide measures of uncertainty, 95% highest probability density intervals (HDI) around the mode were evaluated for parameter estimates.

#### NPP-task specific statistical analysis

For the mechanical pain detection threshold, the geometric mean and the standard deviation were calculated based on the five collected intensities for “Yes” and “No” responses. The statistics were calculated for the test and control arm both before and after the NPP-task.

To validate the method of nociceptive stimulation and test for potential habituation or sensitization effects, we analyzed participants’ responses during the QUEST and main body of the NPP-task. A convergence between the QUEST stimulus staircases was calculated with a difference in intensity values between staircases and chronologically searching for the trial number, at which all subsequent trials yield intensity differences that are in average lower than 5% of the estimated threshold by QUEST.

A Student’s t test was performed to test for differences in QUEST threshold values between female and male participants. A difference in counts between female and male participants with a *ν* > 1 was tested with a chi-square test of independence with a significance test level of 0.05.

To check if the participants were able to detect the intensities of the different trial types during the main body of the task, the detection rate was calculated by dividing the number of perceived trials with the total count of trials for each specific type.

## Results

A total of 70 healthy participants were successful recruited and tested. The NPP-task procedure lasted on average 45 min and was successfully completed by all participants. All participants reported a pricking sensation during electrical stimulation. The analysis of the DES showed no signs of atypical dissociative experiences in our sample (supplementary Fig. S1). Around 13% reported slightly higher anomalous perceptions, scoring high for the CAPS total score similar to average scores of a psychotic inpatients group reported by Bell et al.^33,53^. No correlation could be observed between the DES and CAPS scores and the estimated $\upnu$ or other QST outcomes (see supplementary results for more details).

### Determination of individual stimulus intensities

**Figure 3:**
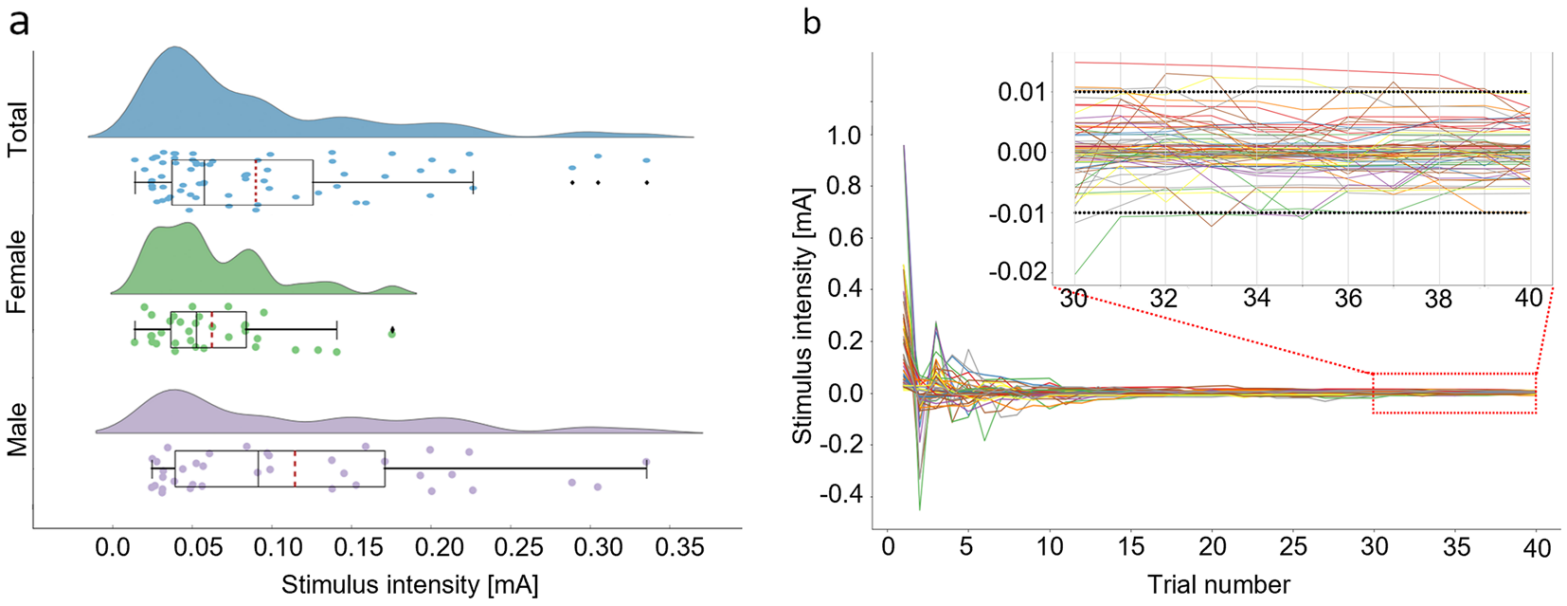
Sensory threshold detection via QUEST. The detection threshold for electrical stimuli was determined by an adaptive maximum-likelihood procedure—QUEST. (**a**) Estimated detection thresholds: Violin plots of detection thresholds estimated by the QUEST procedure for the total (blue), female (green) and male (blue) population. The distribution of threshold intensities is shown both as histograms and in boxplots with medians (back solid line) and average values (red dotted line) and outliers (black dots). Individual intensity values are shown as dots in the color of the respective population. (**b**) Convergence of QUEST stimulus intensities: Difference between stimulus intensities [mA] administered at each trial interchangeably within two independent staircases during the thresholding procedure. The view for the last 10 trials of administered stimuli is enlarged, to showcase a convergence between the staircases within an intensity range between − 0.01 and 0.01 mA. mA—milliamperes.

The average threshold value for the sample, detected by the QUEST algorithm, was 0.09 ± 0.07 mA. The threshold value for males (0.11 ± 0.08 mA) was higher than females (0.06 ± 0.04 mA, *p* = 0.004; Fig. 3a). The differences in currently estimated thresholds between the QUEST staircases converge towards smaller values as the trial number progresses (Fig. 3b) with an estimated average trial number of 21, until all subsequent differences are less than 5% of the estimated detection threshold (0.005 mA).

### Probabilistic Pavlovian conditioning

**Figure 4:**
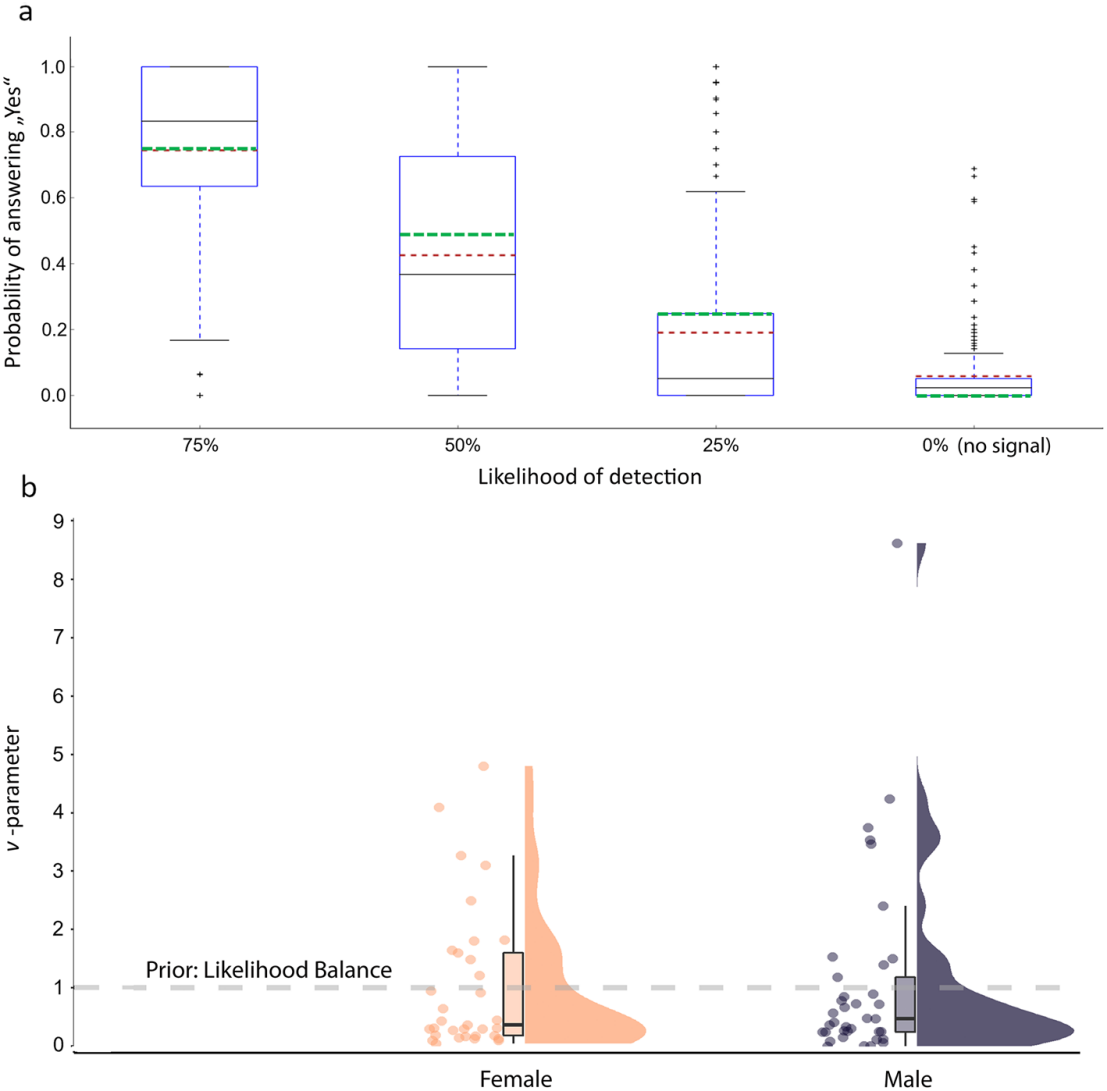
**NPP-task behavioral observations.** Analysis of behavioral data from 70 healthy participants that completed the NPP-task during which the perception of electrical stimuli, associated with varying strengths to a visual cue, is being recorded. (**a**) Trial type detection rates: Box plot of observed participant detection rates for trial types administered during the main body of the task. The x-axis shows the four different stimulus types that were repeatedly administered during the task along with the visual cues. The trial types were characterized by varying intensity values derived from the psychometric curve of each individual participant and respective expected rates of detection of 75%, 50%, 25%, and 0% (green dotted line). The y-axis shows the observed rate at which the participants detected the corresponding trial types with the median (black solid line), mean (red dotted line) values and the individual outlier values (+). (**b**) Weighting between prior and sensory information: Violin plots of the weighting parameter (ν﻿) between prior expectations and sensory evidence during the process of perception extracted by modelling participant’s behavior during the NPP-task with the Hierarchical Gaussian Filter. The distribution of ν﻿ is shown for female (orange) and male (grey) participants. ν﻿ = 1 (gray dotted line) indicates a balance in prior and sensory input weighting (grey dotted line). ν﻿ < 1 and ν﻿ > 1 indicate a higher weighting in sensory input and prior expectations during the process of perception, respectively; NPP-task—Nociceptive predictive processing task.

To test whether participants detected the intensities from the different trial types as expected, their performance based on the detection rate for each type was calculated. Overall, the detection rates were within the expected range according to the psychometric function. Accordingly, detection rates were around 75% (median 80%) for stimulus intensities with an expected 75% uncertainty rate, 43% (median 37%) for stimulus intensities with an expected 50% uncertainty rate, and 19% (median 5%) for stimulus intensities with an expected 25% uncertainty rate (Fig. 4a). Notably, 6% of zero-intensity trials (checkerboard only) were reported as pricking, indicating an effective conditioning procedure.

### Behavioral data and computational modelling

The behavioral data from the individual participants were fitted to the HGF model to extract the parameter characterizing the weighting of prior expectations versus sensory evidence during perception ($\upnu$-parameter). Initially, the $\upnu$-parameter was calculated for each individual. There was a strong tendency for $\upnu$-values to be less than 1 (Fig. 4b). Overall, 30% of participants had a $\upnu$-values greater than one, indicating a stronger weighting of prior expectations over sensory information (34% for females and 26% for males; *p* = 0.46).

Comparison of the mechanical detection threshold between the test arm and the control arm as a possible indicator of local sensitization by the repeated stimulation showed no side differences either before (102.7 ± 63.6 mN and 97.6 ± 63.8 mN, respectively, *p* = 0.63) or after (62.1 ± 35.8 mN and 65.1 ± 38.9 mN, respectively, *p* = 0.63) the NPP task. For the regression model investigating potential changes in mechanical pain detection thresholds from pre to post NPP-task, no evidence for a systematic effect was observed at the test arm or between test arm and control arm. As can be seen in Table 1, the intercept for pre-task thresholds was 7.73 (HDI: 2.72–12.67), whereas for post-task it was 7.40 (HDI: 2.36–12.31), suggesting no differences. All coefficients showed significant overlap with the ROPE. Fit indices indicated that the model explained only little of the variance in $\upnu$ (R_2_ = 0.001), with a LOO-IC of 1645.3 ± 35.3. LOO-cross validation showed that all Pareto k estimates were below 0.5, suggesting that leaving out any one individual observation would not substantially affect the posterior distribution. Rhat and ESS diagnostics suggested good model convergence and reliability of the bulks and tails of the posterior distribution.

**Table 1:** Effect of learning task on the mechanical pain detection threshold pre-postI.

Predictor	Median	SE	95% HDI	ROPE (%)	Rhat	Bulk ESS	Tail ESS
Pre	7.73	2.54	2.72–12.67	97.15	1.00	11,350	10,675
Post	7.40	2.52	2.36–12.31	98.46	1.00	10,962	11,215
Gender (male)	6.59	2.35	1.97–11.16	100.00	1.00	12,698	10,416
QUEST Threshold	1.35	2.52	− 3.63﻿–6.28	100.00	1.00	14,172	11,550
$\sigma$	37.16	6.26	26.06–50.41		1.00	7916	11,031
${\upnu }$(t-dist.)	1.38	0.25	1.03–1.97		1.00	7441	6074

					Bayes R2	LOO-IC	LOO-IC SE
					0.001	1645.3	35.3

*HDI* highest density interval, *ROPE* region of practical equivalence, *Bulk/Tail ESS* bulk- and tail effective sample size, *Bayes R2* R-squared metric for Bayesian regression models, *LOO-IC* leave-one-out cross-validation information criterion, *Rhat* convergence diagnostic for Hamiltonian Monte Carlo sampling.

The model comparison approaches between the “HGF with $\upnu$”, an “HGF without $\upnu$” and a non-Bayesian Rescorla-Wagner model showed a better performance of the “HGF with $\upnu$” in simulating behavioral data, compared to the other models, and a clear support for the “HGF with $\upnu$” based on Bayesian model selection procedures (supplementary Fig. S2). Please see the supplementary material for more details.

### Sensitivity analysis

To explore the relationship of the modeled pain perceptions with the common psychophysical paradigms for determining sensitivity of central pain mechanisms, Bayesian regression analyses were performed with the $\upnu$-parameter values and different QST parameters. For the model estimating the $\upnu$-parameter, none of the predictors showed evidence for an association with the HGF-outcome (Table 2). All coefficients showed significant overlap with the ROPE, with the notable exception of QUEST thresholds. However, the 95% HDI for QUEST thresholds also indicated considerable uncertainty about the parameter estimate. Fit indices indicated that the model explained only little of the variance in $\upnu$ (Bayes R_2_ = 0.01), with a LOO-IC of 211.5 ± 27.4 (see also Table 2). Pareto k diagnostics showed that all but one observation had a value below 0.5, with the remaining observation still falling below 0.7, again suggesting that leaving out any one individual observation would not substantially affect the posterior distribution. Rhat and ESS suggested good chain convergence and reliability of posterior samples.

**Table 2:** Prediction of HGF $\upnu$-parameter.

Predictor	Median	SE	95% HDI	ROPE	Rhat	Bulk ESS	Tail ESS
TSP-effect	0.00	0.14	− 0.25﻿–0.30	76.28%	1.00	10,933	10,392
CPM-effect	− 0.01	0.01	− 0.02﻿–0.01	100.00%	1.00	12,154	9510
Gender (male)	0.04	0.15	− 0.27﻿–0.32	70.25%	1.00	8539	7798
QUEST Threshold	− 0.69	0.98	− 2.64﻿–1.27	9.87%	1.00	10,283	9883
$\sigma$	0.40	0.10	0.24﻿–0.62		1.00	7241	9188
${\upnu }$(t-dist.)	1.27	0.27	1.01﻿–1.99		1.00	6605	6219

					Bayes R2	LOO-IC	LOO-IC SE
					0.01	211.5	27.4

*HDI* highest density interval, *ROPE* region of practical equivalence, *Bulk/Tail ESS* bulk- and tail effective sample size, *Bayes R2* R-squared metric for Bayesian regression models, *LOO-IC* leave-one-out cross-validation information criterion, *Rhat* convergence diagnostic for Hamiltonian Monte Carlo sampling.

## Discussion

The overall goal of this study was to develop a novel sensory testing paradigm that disentangles the influences of neurocognitive expectations and sensory input during pain perception at an individual level. To date, paradigms have been predominantly limited to measuring simple dynamic outputs of central pain processing, such as TSP, CPM, or stress-induced analgesia. In this respect, the presented method focuses not only on the physiological characterization of perception (sensory/pain thresholds), but moreover on the neurocognitive computations in the perception of pain.

The NPP-task was developed to administer pricking electrical stimulations robustly around the detection threshold and induce a strong prior by associating visual cues with a suprathreshold stimulus. Subsequent HGF modelling allowed us to extract the individual influences of prior and sensory information during perception ($\upnu$-parameter). Existing studies regarding involvement of prior information in perceptual decision-making indirectly derive the involvement of the prior from shifted starting points in the perceptual decision-making process during cued pain perceptions tasks^15,54–56^. In contrast, the present paradigm allows a direct estimation of prior weighting within the cognitive computational process during perception modelled within the framework of the HGF. This permits a more concise study of the prior and its link with other learning and decision-making parameters such as the volatility, drift, and learning rate.

Healthy, pain-free subjects show a strong tendency of weighting estimates $\upnu$ < 1, indicating increased weighting of sensory information over prior beliefs. Interestingly, in the smaller subset of participants, the influence of prior beliefs appears to be stronger. This may represent a natural variability of the weighting within a healthy population. Otherwise, one can speculate that this represents a subpopulation with a specific neurocognitive pain processing even potentially representing a special risk group. It is worth noting that, although the value of $\upnu$ = 1 mathematically represents an equal weighting between prior and sensory information, this should not be confused with how “physiological” this is in a given environment; for this, a relative comparison of $\upnu$-values between individuals within the same paradigm is relevant. In the study of Powers et al., the same experimental paradigm was conducted with auditory stimuli. The healthy controls showed a smaller variance in weighting estimates, compared to our sample^35^. This discrepancy in variability might be due to a potentially more salient stimulus type in our study, representing a more direct threat signal and past perception, in form of a prior, is being considered much strictly a factor for precaution. This would explain the higher weighting estimates in our study compared to Powers et al., but not necessarily the large variability within the group. Additionally, we could not find any evidence for an association $\upnu$ with any experiences of dissociation or anomalous perceptions (supplementary results). Around 13% of our participants scored higher in their CAPS total score compared to a psychotic inpatient sample reported by Bell et al.^33,53^. We would argue that this rate is still in line with reports from the general population, where 11% report high levels of anomalous perceptions that are not distressing or compromising^33,53^. These findings suggest that $\upnu$ is not a phenomenon mainly associated with experiences of dissociation or anomalous perceptions. Therefore, the variance of $\upnu$ assessed in our study may still represent a normal variability of a healthy population and the notion of a subpopulation, with high $\upnu$ values implying an aberrant pain perception and acting as a potential risk group, remains rather speculative and further research is needed to clarify this. Overall, it can be stated that the weighting estimates for the participants showed a large range with ν < 1 and others ν > 1, which indicates strong inter-individual differences in pain processing.

The significance of the distinct weighting remains as an important question. The Bayesian regression analysis showed no association between the prior weighting estimates and TSP and CPM. Therefore, NPP-task provides a novel somatosensory biomarker for the study of pain perception, as the weighting estimate $\upnu$ is not associated with known markers of facilitated central pain mechanisms. In most participants, a weighting estimate $\upnu$ < 1, and therefore a higher reliance on sensory information during perception, is as expected. Inference about the causes of nociceptive signals i.e., the “threat” of the world, should incorporate some amount of prior expectation in a normal/non-pathological learner. Nevertheless, the stronger consideration of new sensory information ultimately leads to a more accurate internal model about the new states of the world and thus more efficient learning, especially under changing conditions, (e.g., decrease in association between nociceptive and visual stimuli in our study). In contrast, a potential bias towards prior beliefs, where uncertainty is met with sticking to already known inferential states, leads to perceptions that are not backed up by sensory evidence and therefore new states of the world are not learned accurately. This trait of a stronger prior in pain processing could be associated with higher expectations and higher affectual processing of pain in a clinical sense (e.g., increased pain catastrophizing). The extent of strong prior weighting could further be linked to previous exposure to traumatic events, stress or allodynia and hyperalgesia, all characteristics known to be prevalent and strongly associated with chronic pain syndromes. With respect to this, future studies with chronic pain patients could show if a strong prior during pain perception is associated with trauma, stress and pain catastrophizing, and whether it could potentially cause these subjects to prevail in a “threatful” or “precautious” perceptual state, although sensory evidence would suggest the contrary. A link between a strong prior and varying degrees of perceived pain intensity and chronicity would validate the $\upnu$-parameter as a biomarker of chronic pain and even a potential predictor of chronicity.

Methodologically, there are two important characteristics worth emphasizing: Application of nociceptive stimuli with a given perceptual uncertainty, and modelling of learning trajectories over time. For this, it is of central importance to keep stimuli with a given perceptual uncertainty stable over time. Processes of habituation and sensitization must therefore be prevented. The paradigm presented here ensures this in two ways. Convergence of estimated sensory thresholds appears to be stable within relatively few trials within and between the QUEST staircases. These results suggest that the experimental duration could be reduced, as there are few strong divergences from perceptual thresholds after roughly 20 trials per staircase. A second indicator of stabilized perceptual stimuli can be inferred from the assessment of mechanical pain detection via pinprick. This was carried out on the test and control arm before and after the NPP-task. Estimation of pain detection thresholds suggested no difference from pre- to post-task between the arms. Furthermore, our subjects detect the different trial-type intensities throughout the task with rates as expected from the psychometric curve, with high variability among subjects. Together these findings strongly suggest that there is no evidence of habituation or sensitization across time. Thus, the repeated application of electrical stimuli around a stable perceptual threshold could be relied upon for the learning portion of our task.

Another explanation for the variability in the $\upnu$-parameter could be provided by a prior down-regulation process as suggested in previous studies^57^. It has been shown that participants are less likely to be influenced by a cue that is accompanied by a highly discrepant stimulus^58^. This might indicate a range of uncertainty in the likelihood, where perception is most influenced by the interplay between prior and sensory information. Beyond this, the stimuli are either perceived most of the time or not (highly supra- or sub-threshold stimuli, respectively). Therefore, the 75% and no-signal trials in our study could be that “noticeable” i.e. discrepant, as compared to the other trial types, that we observe a ceiling-floor effect, where prior information is being down-regulated during the cognitive weighting process. It is sensible to assume that this down regulation is highly variable between our participants and could account for the observed variability in the conditioning effect and $\upnu$-parameter.

The key limitations of this study pertain to qualities of the administered stimuli. First, we did not systematically assess participants perceptual qualities of the experienced electrical stimuli. Qualitatively speaking, especially with higher thresholds, participants were often reporting pulse-like perceptions, rather than a pricking. This relates to a second issue—whilst we tuned our stimuli to target A-δ fibers, we very likely caused co-activation of A–β and C fibers too. Moreover, the relative densities of these fibers may vary within participants, as well as by electrode placement on the volar forearm, making repetition of the task under similar conditions extremely difficult. Using a stimulation method selective towards the A-δ fibers and obtaining a better quality of administered stimuli would also help in reducing the observed variability in detection rates for the different trial types. Moreover, it would be worth investigating whether test sites other than the volar forearm show similar perceptual properties, or if each body region has its own characteristic perceptual profile, with distinct influences of top-down cognitive processes. Previous studies on CPM did not show any changes in the position of the conditioning stimulus^59^. It would be worth investigating whether the central processes behind the prior and sensory evidence weighting are also robust across different body regions.

As verified by assessing QUEST convergence and mechanical pain sensitivity before and after the NPP-task, the most notable strength of our paradigm is the stabilization of electrical stimuli to perceptual thresholds across time and repeated stimulations. This ensures that the subsequent learning task measures are a function of neurocomputational processing, rather than an epiphenomenon of localized tissue alterations such as damage to ion channels. Moreover, to our knowledge, our paradigm is the first ever to manipulate uncertainty of sensory input (the varying likelihood of stimulus intensities) in studying the nociceptive system, as previous studies have focused only on uncertainty in the prior^6,60^. This provides another perspective on the modelling of pain perception that is aligned with real-life experiences of noisy and multifactorial environmental contributors to pain. Additionally, our HGF with the $\upnu$-parameter outperformed common non-Bayesian models, like the Rescorla-Wagner, and an “HGF without $\upnu$”. With this, we can endorse the utilization of this Bayesian hierarchical modelling approach with weighting of prior and sensory evidence as a suitable modelling framework for describing the learning in a changing environment under uncertainty for the nociceptive system.

As future work, the electrical stimulation could be optimized for a more selective targeting of nociceptive fibers that would yield in a more nuanced transition of perception of nociceptive sensations over different stimulus intensities and therefore reduce the variability of observed detection rates for different trial types. Another interesting question is whether estimated $\upnu$ values are varying across different body regions (e.g., depending on whether this region has a positive pain history or not). This also applies to exploration of the associations of $\upnu$ with CPM and TSP. In our study, we used different test areas (leg for CPM/TSP versus arm for the NPP-task). Therefore, the exploration of alternative test sites would be helpful to assess whether this may have affected our results. Moreover, it would be interesting to investigate whether $\upnu$ is a trait or rather a state marker, with the former making it a potential predictor for pain chronicity. Although our analysis did not show any association between $\upnu$ and traits associated with dissociative experiences or anomalous perceptions, further investigations of possible links to pathological traits such as pain catastrophizing and anxiety could shed a light onto a common phenomenon of emotional and affective processing and regulation. Finally, the NPP-task and the subsequent modelling of pain perceptions should be undertaken in different clinical populations with chronic pain, investigating a potentially higher prior weighting in these populations compared to healthy subjects. Also, studying $\upnu$ as a marker for chronification could be conducted in studies with populations prior to developing chronicity and once chronicity has emerged.

To conclude, this study demonstrates the importance of predictive processing in pain perception and a high variability in prior weighting, suggesting the demand for an individual-based modelling approach in pain. This novel psychophysical testing paradigm provides a method to calculate the influence of prior sensory information on pain perception and potentially distinguish individuals with increased pain chronicity risk from those without.

## Supplementary Information

Supplementary Information.

## Data Availability

The datasets generated and/or analysed during the current study are not publicly available due to local data protection regulations but are available from the corresponding author on reasonable request. Please contact: jonas.tesarz@med.uni-heidelberg.de for data inquiries.

## Abbreviations

CPM: Conditioned pain modulation
ESS: Effective sample size
HDI: Highest density interval
HGF: Hierarchical Gaussian filter
HMC: Hamiltonian Monte Carlo
LOO-IC: Leave-one-out cross-validation information criterion
NPP-task: Nociceptive predictive processing task
PDT: Pain detection threshold
PTT: Pain tolerance threshold
ROPE: Region of practical equivalence
TSP: Temporal summation of pain
QST: Quantitative sensory testing
