# Supplementary material for: A novel computational approach to pain perception modelling within a Bayesian framework using quantitative sensory testing
Source: Sci Rep. 2023 Feb 23;13:3196. doi: 10.1038/s41598-023-29758-8 (PMC9950064; doi:10.1038/s41598-023-29758-8)
Supplement: Supplementary file 1 — Supplementary Information. [file 41598_2023_29758_MOESM1_ESM.pdf]

Supplementary material

A novel computational approach to pain perception modelling within a Bayesian framework using quantitative sensory testing

Armin Drusko[1^], David Baumeister[1^], Megan McPhee Christensen[2], Sebastian Kold[2], Victoria Lynn Fisher[4], Rolf-Detlef Treede[3], Albert Powers[4], Thomas Graven-Nielsen[2+], Jonas Tesarz[1+*]

[1] Department of General Internal Medicine and Psychosomatics, University Hospital Heidelberg, Germany

[2] Center for Neuroplasticity and Pain (CNAP), Department of Health Science and Technology, Aalborg University, Denmark

[3] Mannheim Center for Translational Neuroscience (MCTN), Heidelberg University, Germany

[4] Department of Psychiatry, Yale University School of Medicine, New Haven, CT, USA.

[^] shared first authorship

[+] shared senior authorship

[*] corresponding author

Address correspondence to:

Prof. (apl.) Dr. Jonas Tesarz, Department of General Internal Medicine and Psychosomatics

Medical Hospital, University of Heidelberg

Im Neuenheimer Feld 410,

D-69120 Heidelberg, Germany

Tel.: +49 6221 56 37862;

Fax: +49 6221 56 5749

E-mail: jonas.tesarz@med.uni-heidelberg.de (JT)

Methods

Dissociative experiences and anomalous perceptions

To assure that perceptual characteristics described by the HGF modelling are not compromised by potential dissociative experiences and/or experiences of anomalous perceptions, we assessed and analyzed the level of occurrence of such experiences and their potential association with outcomes of quantitative sensory paradigms and HGF modelling.

For this, the Dissociative experience scale (DES) and Cardiff anomalous perception scale (CAPS) were assessed in our study sample[1,2]. The DES-score represents the level of dissociative symptoms and is given in a range from 0 to 100. A score above 30 indicates a high level of reported dissociative experiences[1]. The CAPS represents the level of anomalous perceptions along with their distress, intrusiveness, and frequency. The evaluation yields in subscales for the dimensions distress, intrusiveness, and frequency and a total score. The subscales were calculated as the sum of the ratings on their respective dimension. Non-endorsed experiences were set to a score of 0 for each subscale. The total score was calculated as the sum of the endorsed experiences. The CAPS scores are given in a range from 0 to 32, for the total score, and 0 to 160 for the subscales, respectively[2].

To investigate any potential effects of reported dissociation and anomalous perceptions in our sample with the key outcomes, a Kendall correlation was conducted for the DES score and the CAPS total scores and its subscales with the outcomes of the quantitative sensory testing paradigms (pre/post, control/test arm MDT; threshold for electrical stimuli determined by the QUEST algorithm, conditioned-pain modulation (CPM) pain detection (PDT) and pain tolerance (PTT) thresholds; temporal summation (TSP)), and the weighting of the prior versus sensory evidence (ν)[3]. p-values were corrected using the Holm-Bonferroni correction method[4].

Model comparison

In line with literature from previous studies, we compared the performance of the HGF model with the ν-parameter (weighting of prior and sensory evidence), utilized in this study, to non-Bayesian approaches and “HGF without ν”[5,6]. For this, the behavioral data was fit to a Rescorla-Wagner model, an “HGF without ν” and an “HGF with ν”[7]. The ability of the different models to simulate the participants’ behavior was then

investigated by reintroducing the estimated parameters to each respective model and by simulating participants' responses. The simulated and observed responses for each participant were compared by calculating the percentage of identical responses. Furthermore, the Matthews correlation coefficient (MCC) was calculated between the simulation and observed datasets[8,9]. We calculated potential difference in averages of identical responses and the MCCs between the three models by an ANOVA analysis.

Additionally, we conducted a random-effects Bayesian model selection, with the protected exceedance probability (PXP) as the comparison measure, to get an idea about which model would be favored regarding the fit to our behavioral data[10]. The PXP represents the probability that a specific model is more frequent in the population of models that are compared during the BMS.

Results

Dissociative experiences and anomalous perception

The mean DES score for our sample was 1.71±1.3, which is clearly under the cutoff value of 30 and the estimated mean score values across the general population of 7.9 and 3.7, as reported by Carlson et al. and Goldner et al., respectively[1,11]. The mean CAPS total and subscale score for distress, intrusiveness and frequency were 10.9±6.3, 26.3±18.4, 28.8±18.9 and 24±17.9, respectively (Fig. S1).

Approximately 14 % of our subjects had scores for CAPS subscales, and 13 % scores in the CAPS total score, that were higher than their respective mean scores of a psychotic inpatients group reported by Bell et al.[2]

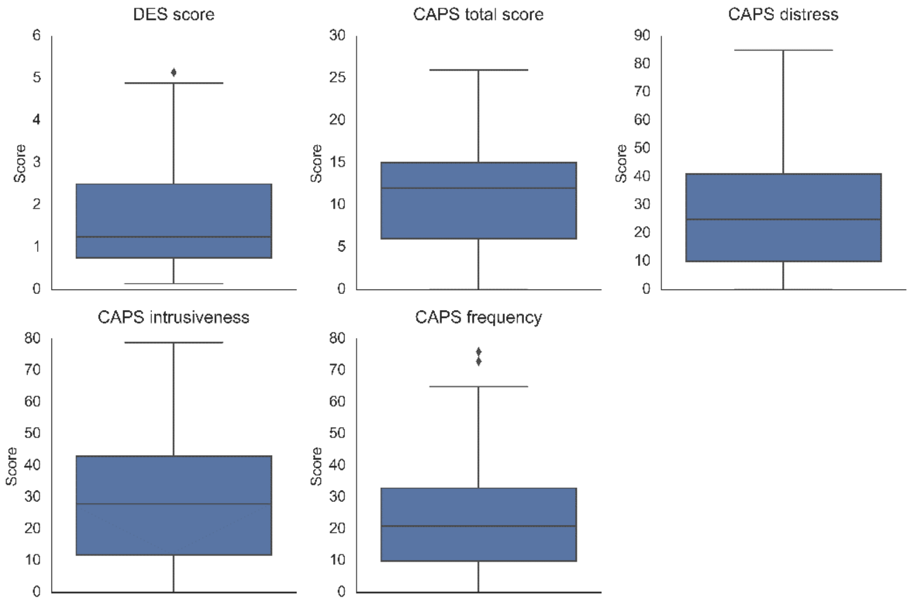

Figure S1: Dissociative experiences and anomalous perceptions*. The level of reported dissociative experiences and anomalous perceptions were assessed by the DES and CAPS questionnaires, respectively. The distribution of the DES total score and the CAPS subscale (distress, intrusiveness, and frequency) and total scores, are presented as boxplots. The DES score values range from 0 to 100. The CAPS total score values range from 0 to 32. The CAPS subscales scores range from 0 to 160. DES – Dissociative experience scale; CAPS – Cardiff anomalous perceptions scale.*

Correlation analysis of the DES and CAPS scores with the QST outcomes and ν did not show any signs of strong association, with the highest correlation coefficient between the ν and the CAPS frequency and total scores being 0.195 and 0.192, respectively (both with a corrected p-value of 1). Table S1 shows the Kendal correlation coefficients for the selected outcomes with DES and CAPS scores.

Based on these results, ν does not seem to be a phenomenon mainly associated with traits of dissociation or anomalous perceptions, as no strong correlations could be found. The proportion of subjects scoring high for CAPS seems to be in line with studies from the general population, where 11% report high level of anomalous perceptions that are not distressing or compromising in any way[2,12].

***Table S1 –** Correlation analysis for DES and CAPS scores with selected QST and HGF modelling outcomes*

DES and CAPS scores	QST and HGF variables	r
**DES total score**	Pre MDT test	-0.031
	Pre MDT control	0.036
	Post MDT test	0.094
	Post MDT control	0.093
	QUEST threshold	0.04
	CPM PDT	0.058
	CPM PTT	-0.002
	TSP	0.043
	ν	0.09
**CAPS total score**	Pre MDT test	-0.097
	Pre MDT control	-0.05
	Post MDT test	-0.037
	Post MDT control	-0.037
	QUEST threshold	-0.171
	CPM PDT	0.035
	CPM PTT	-0.13
	TSP	-0.031
	ν	0.192
**CAPS distress**	Pre MDT test	-0.163
	Pre MDT control	-0.044
	Post MDT test	-0.038
	Post MDT control	-0.011
	QUEST threshold	-0.087
	CPM PDT	-0.017
	CPM PTT	-0.032
	TSP	0.045
	ν	0.147
**CAPS intrusiveness**	Pre MDT test	-0.151
	Pre MDT control	-0.02
	Post MDT test	-0.039
	Post MDT control	0.014
	QUEST threshold	-0.104
	CPM PDT	-0.026
	CPM PTT	-0.057
	TSP	0.011
	ν	0.137
**CAPS frequency**	Pre MDT test	-0.092
	Pre MDT control	0
	Post MDT test	-0.008
	Post MDT control	0.006
	QUEST threshold	-0.094
	CPM PDT	0.002
	CPM PTT	-0.061
	TSP	0.021
	ν	0.195

Note: DES – Dissociative experience scale; CAPS – Cardiff anomalous perceptions scale; QST – quantitative sensory testing; HGF – Hierarchical Gaussian Filter; MDT – pinprick mechanical detection threshold; CPM – conditioned pain modulation; PDT – pain detection threshold; PTT – pain tolerance threshold; TSP – temporal summation effect; ν – perceptual weighting between prior beliefs and sensory evidence; r – Kendall correlation coefficient.

Model comparison

Simulation analysis and Bayesian model comparison between a Rescorla-Wagner model and HGF with and without ν show a clear preference for the HGF model with the weighting of prior and sensory evidence (Fig. S2). The percentages of simulated data identical to the observed, as well as the MCC values, are higher for the HGF with ν compared to the other models. Additionally, the results of the BMS are clearly favoring the "HGF with ν", with higher PXP values compared to the other models. The ANOVA tests for the proportion of identical responses, and the PXP values, showed a statistically significant difference between the three models, ($F(2, 207) = 24.2$, $p = 3.6e-10$) and ($F(2, 201) = 50.17$, $p = 2.1e-18$), respectively.

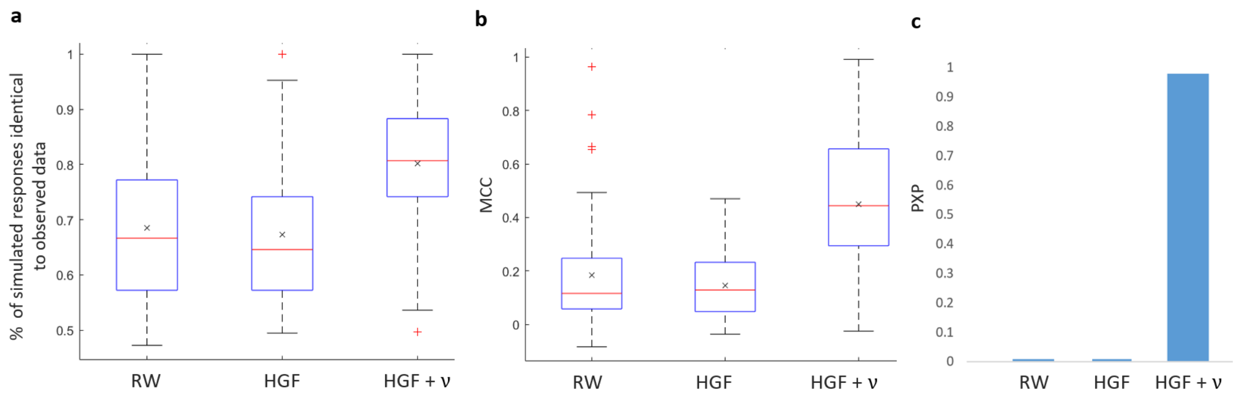

Figure S2: Model comparison. *The HGF model with the weighting of prior and sensory evidence (HGF + ν) was compared with a non-Bayesian Rescorla-Wagner model (RW) and an "HGF without ν" (HGF) in their ability to fit and simulate the observed behavioral data via simulation analysis and Bayesian model comparison. The simulation analysis shows a distribution of* ***a)*** *the percentages of identical responses between simulated and observed data and* ***b)*** *the MCC values for each of the tested models. Boxplots are presented with their interquartile ranges (dashed lines), outliers (red +), median (red lines) and mean (black x) values.* ***c)****The BMS results show PXP values for the tested models; ν – weighting between prior and sensory evidence; MCC – Matthew's correlation coefficient; BMS - random-effects Bayesian model selection; PXP – protected exceedance probability.*

These results demonstrate a superior performance of the "HGF with ν" and point to a good ability to describe and simulate the learning of our participants in contrast to a non-Bayesian model and without ν.

References

1. Carlson, E.B. & Putnam, F.W. An update on the Dissociative Experience Scale. *Dissociation* 6, 16-27 (1993).

2. Bell, V., Halligan, P.W. & Hadyn, D.E. The Cardiff Anomalous Perceptions Scale (CAPS): A New Validated Measure of Anomalous Perceptual Experience, *Schizophr. Bull.* 32, 366-377 (2006).

3. Kendall, M. G. Rank Correlation Methods, London, Griffin (1970).

4. Holm, S. A simple sequentially rejective multiple test procedure. *Scand. J. Stat.* **6**. 65-70 (1979).

5. Powers, A. R., Mathys, C. & Corlett, P. R. Pavlovian conditioning-induced hallucinations result from overweighting of perceptual priors. *Science.* **357**, 596–600 (2017)

6. Kafadar, E., Mittal, V.A., Strauss, G.P., Chapman, H.C., Ellman, L.M., Bansal, S., Gold, J.M., Alderson-Day, B., Evans, S., Moffatt, J., Silverstein, S.M., Walker, E.F., Woods, S.W., Corlett, P.R., Powers, A.R. Modelling perception and behavior in individuals at clinical high risk for psychosis: Support for the predictive processing framework, Schizophr. Res. 226, 167-175 (2020)

7. Rescorla, R.A., Wagner, A.R. A theory of Pavlovian conditioning: Variations in the effectiveness of reinforcement and nonreinforcement. In: Classical Conditioning II: Current Research and Theory (Eds Black AH, Prokasy WF) New York: Appleton Century Crofts, 64-99 (1972)

8. Guilford, JP. Psychometric Methods. New York City: McGraw-Hill, (1954).

9. Chicco, D. & Jurman, G. The advantages of the Matthews correlation coefficient (MCC) over F1 score and accuracy in binary classification evaluation. *BMC Genomics* **21**, 6 (2020).

10. Rigoux, L., Stephan, K.E., Friston, K.J., Daunizeau, J. Bayesian model selection for group studies - Revisited. *NeuroImage* 84, 971-985 (2014)

11. Goldner, L.M., Cockhill, L.A., Bakan, R. & Binningham, C.L. Dissociation experiences and eating disorder. *Am. J. Psychiatry* 148, 1274-1275 (1991).

12. Bell, V., Halligan, P.W., Pugh, K., Freeman, D. Correlates of perceptual distortions in clinical and non-clinical populations using the Cardiff Anomalous Perceptions Scale (CAPS): Associations with anxiety and depression and a re-validation using a representative population sample. *Psychiatry Res.* 189, 451-457 (2011)
